# Individual-level patterns of resource selection do not predict hotspots of contact

**DOI:** 10.1186/s40462-023-00435-9

**Published:** 2023-11-30

**Authors:** Anni Yang, Raoul Boughton, Ryan S. Miller, Nathan P. Snow, Kurt C. Vercauteren, Kim M. Pepin, George Wittemyer

**Affiliations:** 1 Department of Geography and Environmental Sustainability, University of Oklahoma, Norman, OK 73019 USA; 2 Archbold Biological Station, Buck Island Ranch, Lake Placid, FL 33852 USA; 3 United States Department of Agriculture, Animal and Plant Health Inspection Service, Veterinary Service, Center for Epidemiology and Animal Health, 2150 Centre Avenue, Fort Collins, CO 80526 USA; 4 United States Department of Agriculture, Animal and Plant Health Inspection Service, National Wildlife Research Center, Wildlife Services, Fort Collins, CO 80521 USA; 5 Department of Fish, Wildlife and Conservation Biology, Colorado State University, Fort Collins, CO 80523 USA

**Keywords:** Resource-driven contact, Resource selection, Spatial contact, Wild pigs

## Abstract

Contact among animals is crucial for various ecological processes, including social behaviors, disease transmission, and predator–prey interactions. However, the distribution of contact events across time and space is heterogeneous, influenced by environmental factors and biological purposes. Previous studies have assumed that areas with abundant resources and preferred habitats attract more individuals and, therefore, lead to more contact. To examine the accuracy of this assumption, we used a use-available framework to compare landscape factors influencing the location of contacts between wild pigs *(Sus scrofa)* in two study areas in Florida and Texas (USA) from those influencing non-contact space use. We employed a contact-resource selection function (RSF) model, where contact locations were defined as used points and locations without contact as available points. By comparing outputs from this contact RSF with a general, population-level RSF, we assessed the factors driving both habitat selection and contact. We found that the landscape predictors (e.g., wetland, linear features, and food resources) played different roles in habitat selection from contact processes for wild pigs in both study areas. This indicated that pigs interacted with their landscapes differently when choosing habitats compared to when they encountered other individuals. Consequently, relying solely on the spatial overlap of individual or population-level RSF models may lead to a misleading understanding of contact-related ecology. Our findings challenge prevailing assumptions about contact and introduce innovative approaches to better understand the ecological drivers of spatially explicit contact. By accurately predicting the spatial distribution of contact events, we can enhance our understanding of contact based ecological processes and their spatial dynamics.

**Supplementary Information:**

The online version contains supplementary material available at 10.1186/s40462-023-00435-9.

## Introduction

The spatial distribution of direct contact among animals (co-location of animals at the same time) greatly influences the dynamics of various ecological processes, such as disease transmission, social organization, and human-wildlife conflict [12, 19, 26]. The spatial distributions of contacts across landscapes are often heterogeneous due to various factors, including animal movement patterns (e.g., migration, speed), social behaviors (e.g., mating, territoriality, fission–fusion dynamics), densities of animals, and external factors such as food availability and predators [15, 37]. Assessing drivers of such heterogeneity is key for mechanistic understanding of contact-driven ecological processes.

While social factors like group membership can influence contact structure and heterogeneity (i.e., animals within the same social groups tend to stay together, resulting in a higher contact rate) [34], landscape features like the distribution of resources also impact contact patterns [24]. Areas with abundant resources or preferred habitats may attract more animals, including from different social groups, structuring contacts. Exploring the role of the landscape in structuring contacts can facilitate understanding of the spatial distributions and extents of contact based ecological processes and enable the prediction of contacts or contact-driven ecological processes on the landscape.

Frameworks to investigate environmental drivers of contacts and probabilistically predict their distributions are limited. Koen et al. [18] adopted social network analysis to estimate the effects of landscape connectivity on contact rates by comparing contact rates based on broad-scale environmental conditions. In addition, some spatial models (e.g., conditional autoregressive model) have also been applied to quantify the effects of different factors on the spatial distribution of contact rates [41]. However, these approaches have not explicitly compared the spatial conditions where contacts occurred to those where contacts could have occurred. As such, these approaches did not estimate and disentangle how different landscape features drive probability of contact occurrence across the landscape. Building off the assumption that contacts differentially occur at the places with abundant resources or preferred habitat, previous studies have used the spatial overlap of individual or population-level habitat selection as a proxy for contact probability to estimate inter-specific interactions [32], model disease transmissions [22], or simulate contact distributions [13]. However, it remains unclear whether patterns of resource selection and, specifically, the overlap of habitat selection can accurately predict hotspots of contact. Also, it remains unclear whether contact patterns and habitat selection patterns by animals are driven by the same resources.

In this study, we developed a method to quantify landscape factors influential to contact locations. We applied the method to movement data from two wild pig (*Sus scrofa*) populations in dissimilar landscapes, exemplifying how our approach can identify geographical attributes associated with contact events. We use our framework to test the hypothesis that the landscape variables that drive contacts are the same as those that drive individual-level space use, as has been assumed in previous applications of resource selection analysis to contact behavior. Consequently, we assess the utility of aggregating individual resource selection functions to predict the spatial properties of contact behavior. We discuss the implications of this work for understanding contact dynamics in wild pig systems.

## Materials and methods

### Modeling the resource-driven contacts and comparing with individual-level resource selection

To test the hypothesis that the landscape features driving contacts are the same as those that drive individual-level space use, we apply a resource selection functions (RSF) framework to contact locations of animal pairs (hereafter: contact-RSF model) and compared it with the habitat selection RSF of the individual animals involved in a contact pair (individual-RSF model).

#### Modeling preparation

As with RSF models, the data that are needed to develop the contact-RSF model include animal GPS tracking data and landscape features or environmental factors. The location of where direct contacts occurred for each contact pair, i.e., the pair of animals that have contact, was determined from spatial overlay between GPS location data. Several methods have been developed to estimate spatial-explicit contacts based on telemetry data, including Noonan et al. [28], Long et al. [20], Yang et al. [43]. Here we expand on these methods, implementing the continuous-time movement model (CTMM)-contact to estimate missing contacts [40, 43].

#### individual-RSF model for contact pairs

Since the habitat selection of contact pairs are considered as the reference to compare with to test our hypothesis, we first developed an individual-level RSF model for all contact pairs and aggregated them to estimate the spatial distributions of their habitat selection. Before the development of individual-RSF models, we subsampled movement data for each contact pair so as to only include the time period when both animals were tracked, as an animal may change the area used over time. We then combined the subsampled movement data for individuals involved in each contact pair. This allowed time matched comparison with the contact-RSF model predictions aggregated by all contact pairs (Additional file 1: Figure S1).

**Fig. 1:**
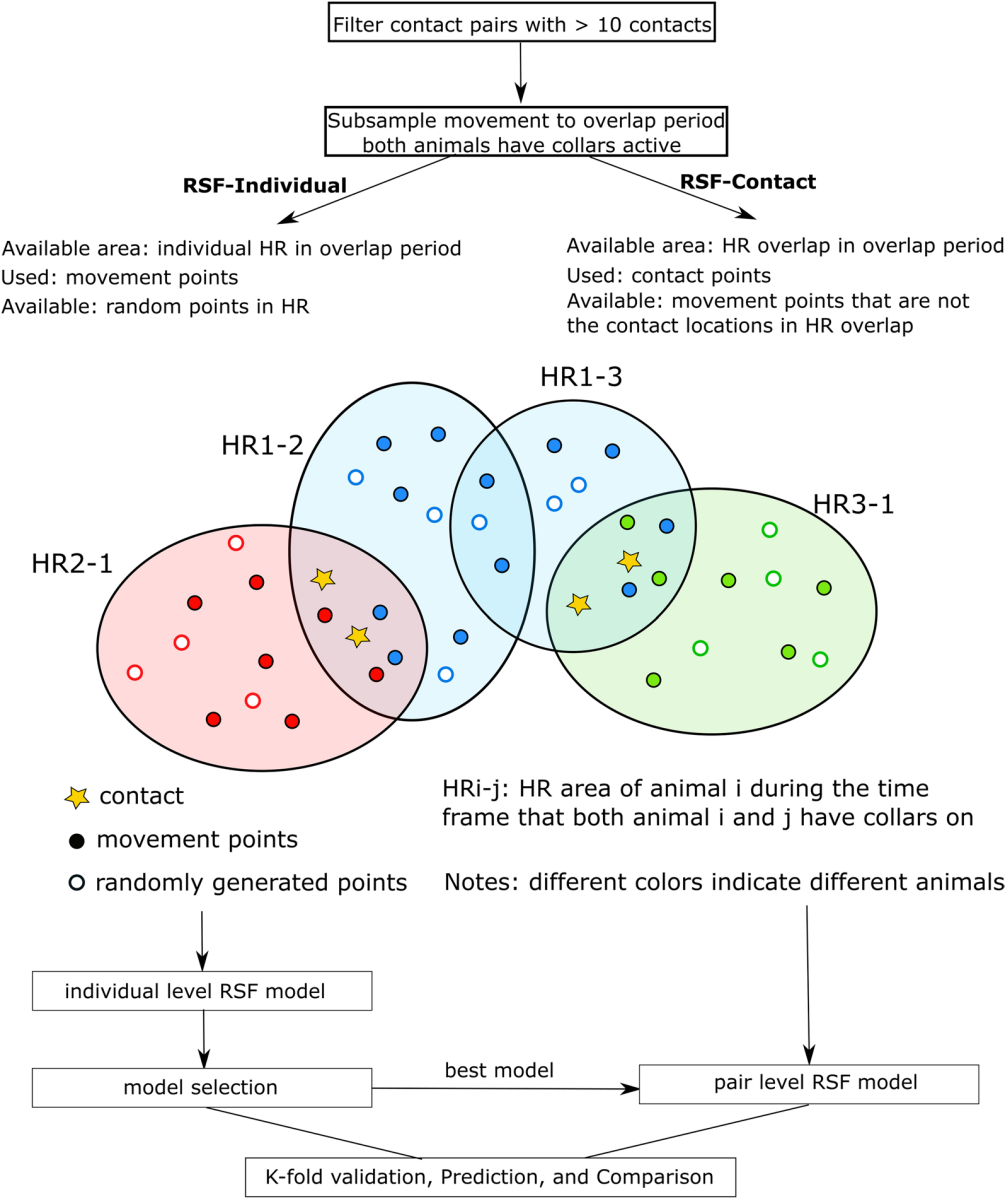
Schematics of individual and contact RSF model approaches

We applied the used-available framework, as described in Manly et al. [23], to develop individual-RSF models for each individual involved in each contact pair. Specifically, the available area is defined as the 95% home range for each individual in the contact pair for the period when movement data collection overlapped with another in the pair (i.e., individual availability at pair level, e.g., HR1-2, HR1-3 in Fig. 1). We defined used points as the interpolated GPS fixes and generated 30 random (available) points per used point within the individual’s home range [29].

For the development of RSFs, we conducted logistic regressions [23], and implemented a model selection procedure to evaluate candidate models using the cumulative log-likelihoods for all animals to calculate Akaike information criteria (AIC) and select the most parsimonious model [3, 8]. We then calculated the population average and confidence intervals of coefficients by weighting both the number of times that each individual is detected in a unique contact pair and their sample size following Murtaugh [27]. We assessed the predictive power of the top-selected model by performing a fivefold cross validation to calculate the Spearman rank correlation coefficient (r_s_) for each individual. We withheld 20% of the data, assessed the model fit, and repeated the process five times for each individual [6]. Development of the individual RSF models was conducted using “IndRSA” R-package [3].

#### Pair-level contact-RSF model

To estimate the RSF for contact, we adopted a similar use-available framework but define the location of contact for each pair of individuals as the used points. We define the available area for contact as the home range overlaps between a pair during an overlapping tracking period. The available points in contact-RSF model are defined as the GPS fixes that were not contact locations within the home range overlaps. For pairs that have numerous available points, a subsample of available points was randomly selected to limit the used: available points ratio to 1:30. We used logistic regression for each contact pair to compare the landscape features at the location of contact with those at locations that animals used without contact.

To test the hypothesis that contact RSFs are similar to individual-level RSFs, we assume the individual-RSF model is the null hypothesis and reference to be compared to. Thus, we fit the logistic model for contact-RSF using the same model structure as that of the top-selected individual-RSF model. Similarly, we follow Murtaugh [27] to aggregate the pair-wise contact-RSF models to a weighted population average to predict the spatial distribution of the occurrence of the resource-driven contact for the animal population on the landscape. Finally, we compare the weighted population-level contact-RSF prediction and the weighted population-level individual RSF to examine whether predictions of resource-based contact are equivalent to individual-RSF patterns. Such use-available design (i.e., use as contact, available as selected locations without contact) and modeling frameworks (i.e., individual RSF models as baseline model) aims to directly test our hypothesis by comparing how resources drive contact and habitat selection.

### Case studies

We implemented the framework of modeling the resource-driven contacts and comparing with individual-level resource selection in two wild pig populations in two different ecosystems. Wild pigs, are a socially structured species that maintain matrilineal, multigenerational social groups of female adults with their offspring [31]. These groups spend most of their time moving together as a unit. Male adults often move alone but join female groups for short periods. Based on these behaviors, we examined contact between individuals from different family groups or between adult males and females because these events are distinct and rarer compared to the many within group contacts and potentially independent of individual-level resource selection.

#### Global positioning system (GPS) data

Our case study includes two sites, one on the Archbold’s Biological Station—Buck Island Ranch (ABIR) in Florida and one on a private ranchland in north-central Texas. ABIR is a 42.3 km^2^ commercial beef cow-calf operation managed at commercial production levels with an average standing inventory of ~ 3,000 head of cattle. In FL, we deployed GPS collars (Catlog GPS device and Lotek LMRT3 VHF Collars, Lotek ©, WA, US) on 17 adult wild pigs (12 females and 5 males) from Dec 13, 2019–July 13, 2020. During the capture, we intended to cover most social groups of the wild pigs across the focal pastures (given pre-collaring camera survey) and avoid deploying multiple collars in the same social group. Such study design aimed to measure between-group contacts to understand potential disease transmission in the population [41]. Collars were programmed to record GPS fixes every 10 min with locational errors of 6–10 m on average. In coordination with these collar deployments, anthropogenic cattle feed and water troughs within the study pastures were mapped and time available recorded.

The Texas site is ~ 52 km^2^ and located within Southwest Plateau and Plains Dry Steppe and Shrub ecoregion of North America, with vegetation communities dominated by a mosaic of wheat croplands, grasslands, mesquite, and oak woodlands [2]. We deployed GPS satellite-transmitting collars (VERTEX PLUS-2 Collar, VECTRONIC Aerospace GmbH, Berlin, Germany) to 36 adult wild pigs (22 females and 14 males) during Jan 2018. We programmed the GPS collars to record locations every 15 min from Jan 28–Feb 24, with locational errors of 5–10 m. The study was initially designed to estimate the efficacy of toxic baiting on controlling wild pigs, therefore we deployed bait sites targeting the collared animals. Starting on Feb 13, the baiting was commenced with whole-kernel corn at a maximum baiting density of 1 bait site per 0.75 × 0.75 km^2^. This grid size was selected to expose 90–100% of wild pigs to bait within the study area. Given the behavioral and movement changes of wild pigs after exposed to toxicants, we only used data collected during the period before deploying toxicants. See further details about study site and design in Texas in [35].

#### Environmental variables

The environmental factors that we tested related to contact occurrence in two wild pig populations are presented in Table 1. For both study sites, the *wetland* variable is a binary layer, with freshwater emergent wetland and woody wetland classified as 1, and all other land cover types classified as 0. Similarly, the *water* variable is a binary layer, with freshwater pond, riverine, lake, and water trough classified as 1, and all other land cover types classified as 0. Vegetation greenness was measured using the daily Normalized Difference Vegetation Index (*NDVI*) accessed from NASA's MODIS MOD09GA product. Tree canopy cover and daily meteorological measurements were accessed from the U.S. Forest Service tree canopy cover product and GridMET, respectively. In the FL site, we also included binary variables for *road, ditch, fence*, and *food*, indicating whether a grid cell includes a road, ditch, fence, or cattle supplement. Similarly, in TX we included binary variables for *road* and *trail (e.g., 2-track road)*, and the *food* layer represents the availability of pig baits. The variable *cattle* in the FL site measures the daily cattle density on each pasture. All environmental layers were calculated or resampled to 30 * 30-m grids.

**Table 1:** Descriptions and sources of environmental variables used in resource selection models

Variables	Descriptions	Sources
*Dynamic daily variables*		
Tmax	Daily maximum temperature	GridMET
Tmin	Daily minimum temperature	
Trange	Daily difference of temperature	
Vp	Daily vaper pressure	
Prcp	Daily precipitation	
Rhum	Daily average relative humidity	
NDVI	Daily normalized difference vegetation index	MODIS MOD09GA products
Food	Daily binary layer of artificial food availability (i.e., cattle supplements in the FL site, pig baits in TX sites)	Field records
*Static variables*		
Water	Binary layer of surface water body (i.e., freshwater pond) and large ditches that might hold water after rainfalls	Field data and National Wetlands Inventory
Wetland	Binary layer of wetland land cover	
Road	Binary layer of road	Field data and Texas Department of Transportation
Fence (FL only)	Binary layer of fence	Field data
Ditch (FL only)	Binary layer of ditches	Field data
Tree canopy	Percentage of tree canopy	NLCD
Trail (TX only)	Binary layer of trails	Digitalized based on satellite image

#### Extract resource selection of contacts based on a continuous-time movement model of GPS data

We converted the movement data from both study areas to continuous-time movement trajectories discretized to 5-min intervals using the “ctmm” R-package [9]. Specifically, we fitted independent identically distributed, Ornstein–Uhlenbeck (OU), Integrated OU, and OU Foraging movement models to each individual and select the best model based on AIC. The interpolation was predicted based on the top-selected model. The 5-min time frame was chosen based on evidence that wild pigs have small home ranges and low rates of daily movement (Kay et al. 2017). We followed Yang et al. [43] to define direct contact as the colocation of two individuals at the same time with a spatial buffer of 10 m to consider the GPS locational errors. Such a continuous-time movement model (CTMM)-contact method was found to recover most missing contacts in discrete movement data.

Because wild pigs in the same group do not move independently, we expected that contact locations among them would track their movement patterns and thus not test our hypothesis. Since only adult wild pigs were studied, we assumed that all female-male and male-male contacts to be between-group contacts which may be driven by reproductive processes and landscape features. For female-female contacts, we examined the weekly home range core area overlap using kernel density home range estimator to exclude pairs that were in the same family group. We assumed that pairs with core area overlaps less than 0.5 over the tracking period were between-group pairs, while pairs with core area overlaps greater than 0.5 were considered within-group pairs. For pairs with core area overlaps over 0.5 for part of the subsequential tracking period (e.g., more than 12 weeks, a season), we assumed that the pairs were temporarily in the same social group. We included all pairs or timeframes of pairs (female-female, female-male, and male-male) that were considered separate groups at each site in the following analyses.

#### Application of contact-RSF modeling framework and comparison with individual-RSF

To ensure reliable statistical inference, we only included the wild pig pairs with more than 10 direct contacts over the time period that movement data from each individual overlapped. Following the modeling framework, we first fit the individual-RSF models for each individual in the contact pairs. The available areas were defined as the 95% home range estimated by the kernel density estimator. The used and available points were defined as CTMM interpolated points and randomly generated points at a ratio of 30 per used points, respectively. We screened environmental variables for multicollinearity (Pearson's correlation coefficient |r|≥ 0.6) and standardized the continuous variables (i.e., NDVI, tmax, tmin, vp, rhum, prcp, and tree canopy) using the scale function in R [33], before the development of logistic regression for individual-RSF models. For contact-RSF models, we defined contact locations as used points and available points as the CTMM interpolated movement locations that were not the contact locations within the home range overlaps. We then followed the modeling framework described above to implement analyses for modeling, scaling up, and validating both individual- and contact-RSFs in the empirical systems.

## Results

### Direct contacts at both sites

We identified 15 and 27 between-group female pairs in FL and TX, respectively, that made more than 10 contacts during the study period. On average, these pairs had 236.3 (± 615.7) and 245.4 (± 426.7) direct contacts, respectively. In the case of female-male pairs, we observed 28 pairs in FL and 38 pairs in TX that had an average of 445.1 (± 485.5) and 280.7 (± 400.1) contacts during the study period, respectively. Additionally, there were 5 and 9 male pairs in FL and TX that exceeded 10 contacts during the study period. These male pairs had an average of 87.25 (± 63.6) and 87.8 (± 134.9) direct contacts, respectively.

### Individual and contact RSF results

Overall, our findings in two wild pig populations suggested that the landscape predictors (e.g., wetland, linear features, and food resources) played different roles in habitat selection and contact processes. The spatial overlap of individuals’ habitat selection does not adequately represent the spatial distribution of contacts across the landscape.

#### Female-female pairs

**Fig. 2:**
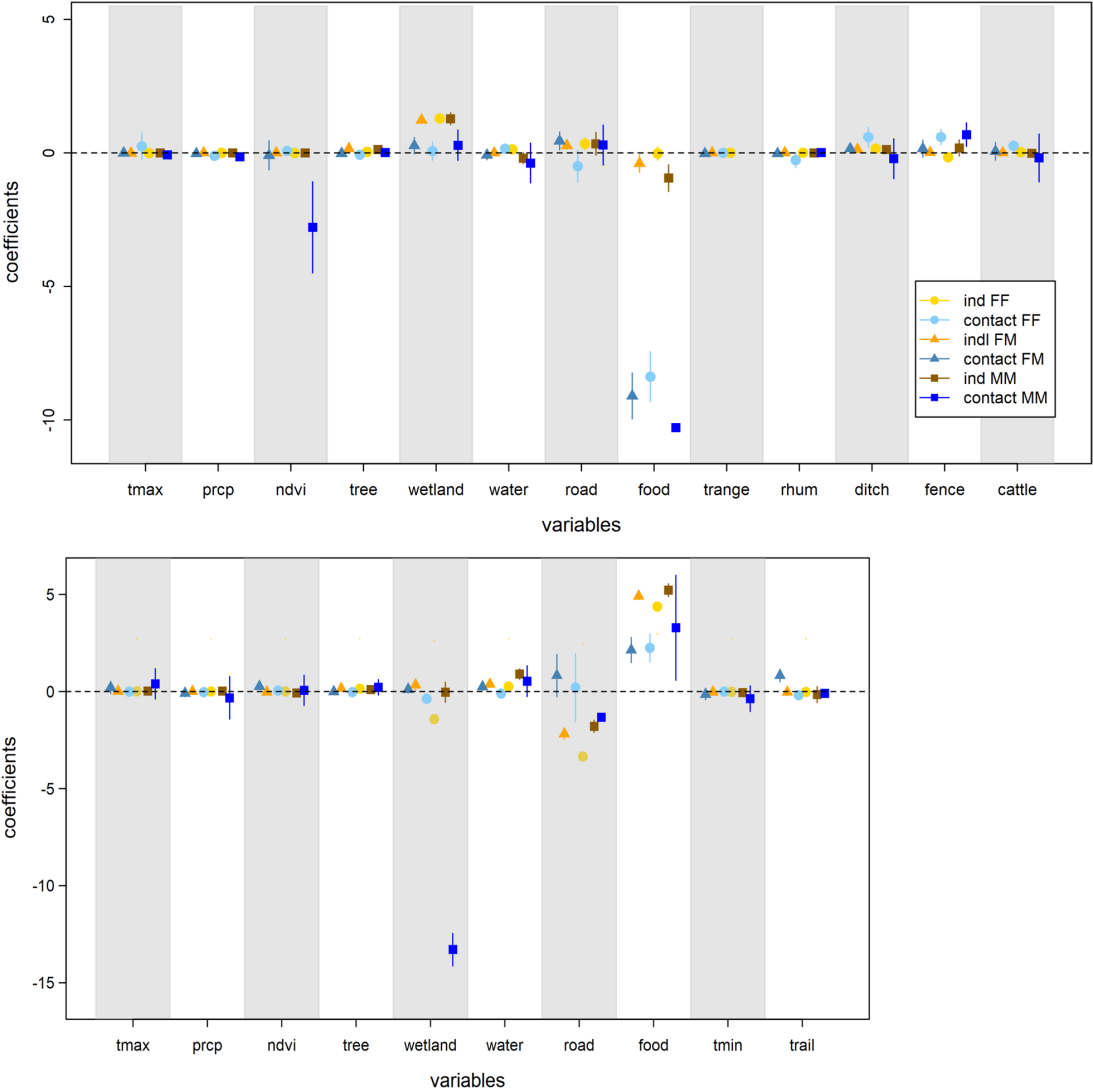
Weighted population-level coefficients of individual and contact RSF models. The top panel corresponds to FL and the bottom panel to TX. See detailed coefficient ranges in Additional file 1: Table S2

**Fig. 3:**
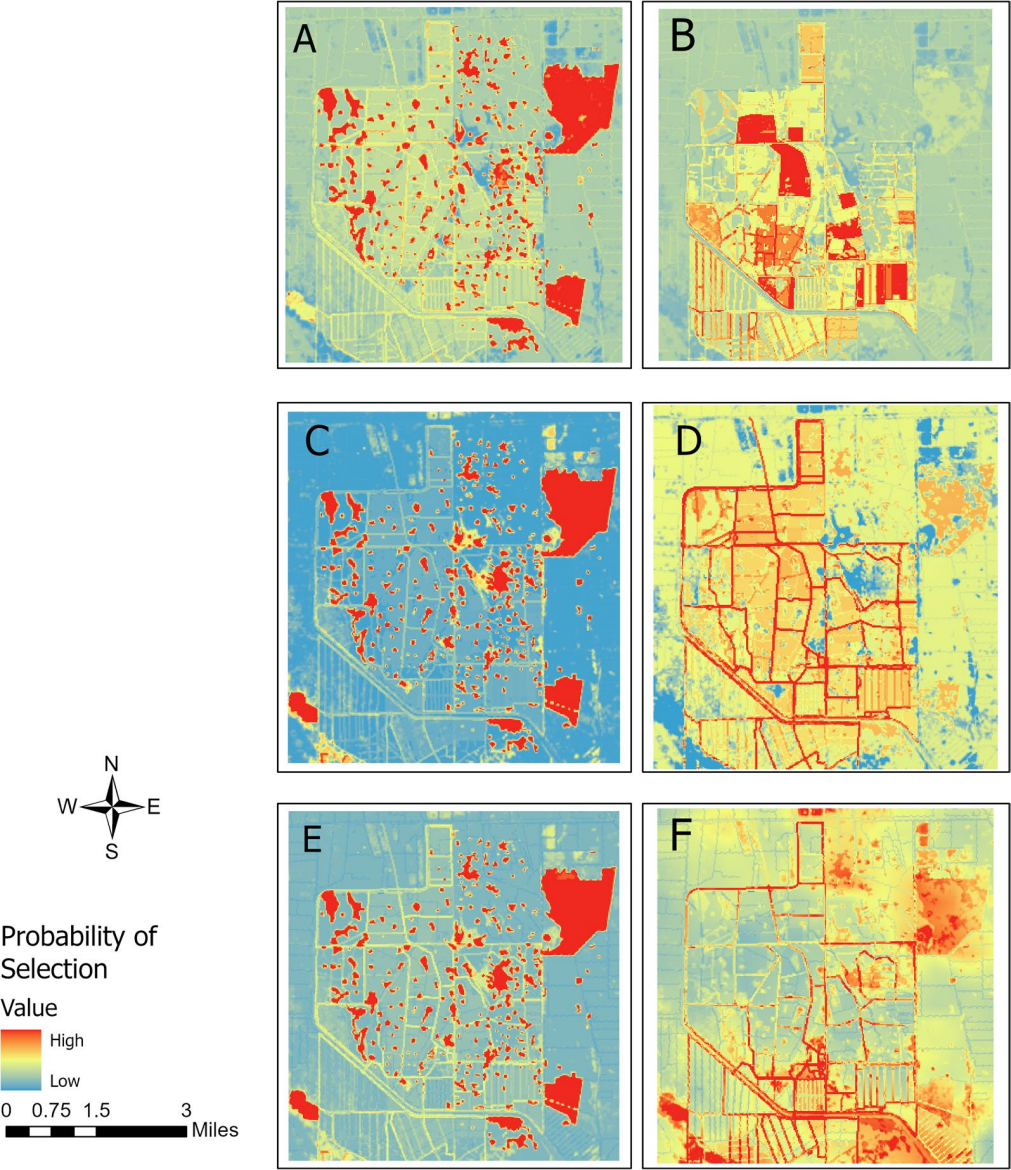
Spatial prediction of population-level resource selection and contact in FL site. **A** habitat selection for FF pairs; **B** contact for FF pairs; **C** habitat selection for FM pairs; **D** contact for FM pairs; **E** habitat selection for MM pairs; **F** contact for MM pairs

In the FL site, models for female wild pig pairs showed selection for wetland, ditches, and water and avoidance of fences (Fig. 2). The top-selected individual-RSF model (Fig. 2) showed wetland areas were the most strongly selected of the resources assessed. Their contacts tended to happen less at food resources, suggesting use of food resources was subject to spatial or temporal segregation. Contacts occurred more frequently along linear features (ditches and fences) and in pastures with higher cattle density relative to their selection of these landscape features (Fig. 3).

**Fig. 4:**
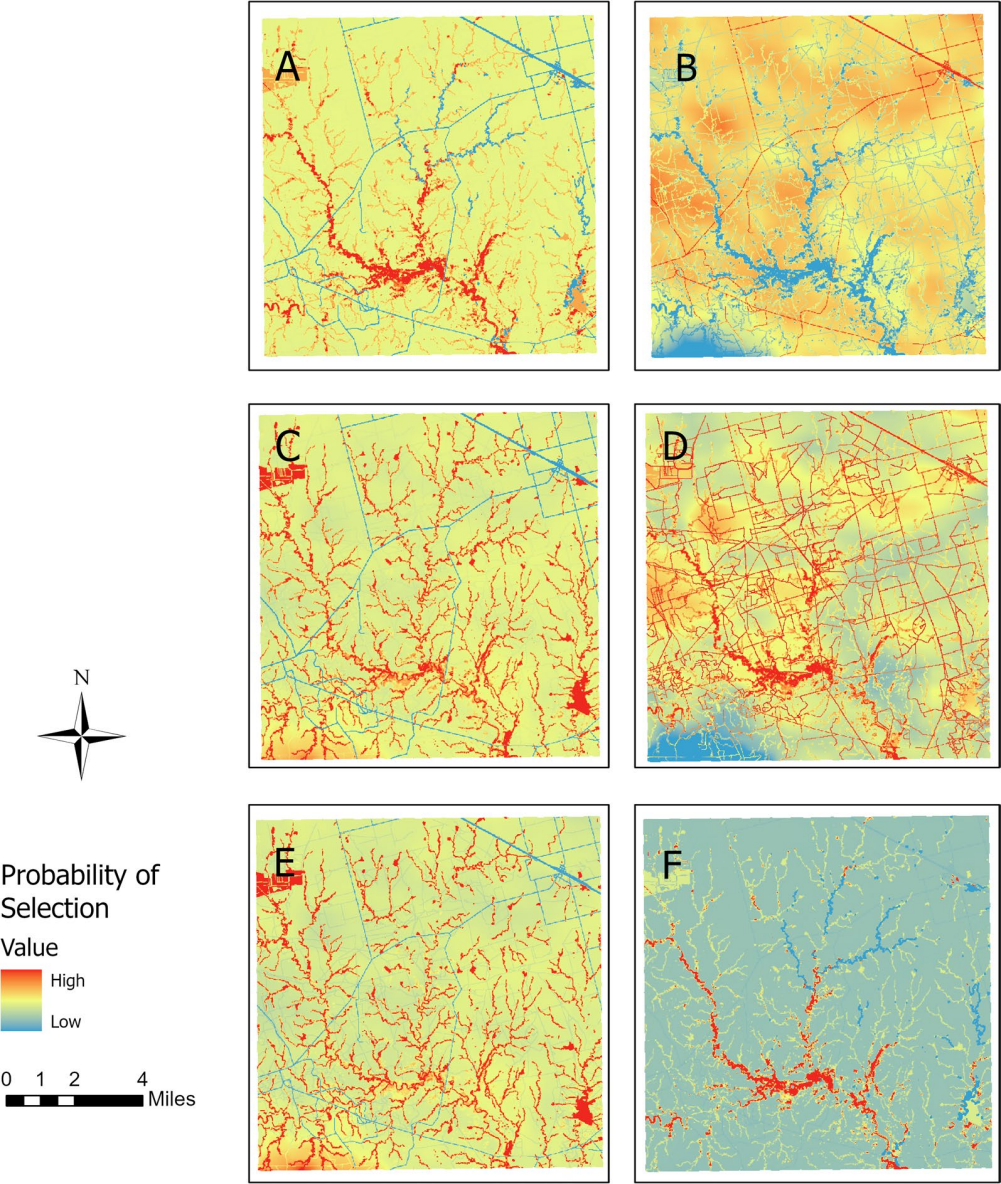
Spatial prediction of population-level resource selection and contact in TX site. **A** habitat selection for FF pairs; **B** contact for FF pairs; **C** habitat selection for FM pairs; **D** contact for FM pairs; **E** habitat selection for MM pairs; **F** contact for MM pairs

In the TX site, female wild pig pairs selected for water and bait sites and avoided wetlands and roads (Model 1.1 in Additional file 1: Table S2; Fig. 2), which resulted in strong habitat selection for riverine areas and low selection along primary roads (Fig. 4). In contrast, their contact locations occurred more at bait sites and less at wetland and water areas, relative to their selection for these features.

#### Female-male pairs

In the FL site, individuals involved in female-male contacts selected for areas with dense tree canopy, wetlands, and locations along ditches and roads, but avoided areas with cattle supplements (Model 2.1 in Additional file 1: Table S1; Fig. 2). Prediction of the individual-RSF model across the landscape showed a high probability of selection for these individuals in wetland areas. Similar to female-female contacts, female-male contacts tended to occur along linear features such as ditches and roads. However, these contacts tended not to be cattle supplements.

In the TX site, individuals involved in female-male contacts selected areas near wetlands, water, and with dense tree canopy and bait availability, but avoided primary and secondary roads (Model 2.1 in Additional file 1: Table S2; Fig. 2), which was reflected in the prediction map (Fig. 3). Contact between female-male pairs was more likely to occur in areas with high NDVI, along trails, and at bait sites.

#### Male-male pairs

In the FL site, male wild pigs selected wetland and high tree canopy areas and avoided cattle supplements (Model 3.1 in Additional file 1: Table S1; Fig. 2). However, their contacts happened more than expected along fences but less at places with high NDVI and cattle supplements, as shown in Figs. 2 and 3. In the TX site, males selected water areas and bait piles for resources and avoided roads. For male-male contact in TX, the individual-RSF was a relatively good approximation of contact RSF, but males tended to make contact at bait sites more than habitat selection and less at roads (Figs. 2 and 3).

## Discussion

Understanding the temporal and spatial processes driving contact among animals can provide insight to factors structuring a myriad of contact-based ecological processes. To this point, assessment of contact structure has generally relied on simple assumptions (e.g., high habitat resource selection area equates to high contact areas) that are often not tested. Here we assessed how well the spatial distribution of contacts matched that of general space use in two feral pig populations inhabiting distinct ecological areas. Our findings highlight that the spatial overlap of individuals (population-level RSF model outputs) does not adequately represent the spatial distribution of contacts across the landscape, which challenges the utility of predictions of contact rates and spatial dynamics of contact dependent ecological processes based solely on RSF outputs. Specifically, landscape features that drive contact between individuals were generally different from those that drive individual-level space-use (Additional file 1: Figure S2 and S3) in two different systems. This suggests differences in the way some factors influenced how wild pigs’ interface with their landscapes compared to how they interact with conspecifics in the areas they inhabit.

### Overlap of habitat selection does not predict spatial distributions of contact

By comparing the contact-RSF model with a paired (overlap) individual-RSF model, we were able to reject our null hypothesis and found that landscape features impacted habitat selection and contact differently. These results indicate that interactions among unmeasured factors such as social behaviors, resource value and competition structure contact and conspecific interactions in more complex ways than purely selection of habitat.

The mismatch between habitat selection behavior and the spatial distribution of contacts has ramifications for our interpretation of contact based ecological processes. In disease systems, accurately predicting the spatial distribution of host contacts is crucial for assessing the risk of spillovers and estimating disease spread. Previous research often used the distribution of host population habitat selection as a proxy for contact to estimate transmission hotspots. For example, in the chronic wasting disease system, overlapping areas that are shared by deer are considered high probability areas for contact and disease transmission, and were used to inform disease control and surveillance priorities [39]. In predator–prey systems, the prediction of predation risk for prey species, such as the probability of encounter, attack, and kill, is often estimated based on the perception of risk by prey in their resource selection or by directly assessing landscape characteristics where predation occurs [1]. However, because landscape features may influence habitat selection and contact differently, estimation of spatial features influencing contact would be more accurate if done directly.

The Contact-RSF model in this study was developed based only on direct contacts (i.e., animals located at the same locations at the same time). The processes underpinning differences in landscape features that drive direct contacts versus individual resource selection could be related to intraspecific competition. When animals approach each other, conspecific attraction, competition, or territoriality can trigger changes in behaviors, which might cause proximity based changes in resource selections [10].

Contacts can also occur indirectly with temporal segregation (i.e., indirect contact: co-location at different times). Yang et al. [43] described the relationship between direct and indirect contacts and highlighted they are not dichotomic processes. In cases of indirect contacts with relatively short temporal segregation, there can still be discernible impacts on subsequently arriving individuals. Cues or signals left by previous visitors may persist in the environment for some time and be perceived by subsequent animals, influencing their behavior. However, in scenarios where no cues are left, we anticipate that the occurrence of indirect contact will align with the overlap in resource selection between the animals.

### System specific biology

In the wild pig system, pigs exhibited a higher probability of contact at locations along certain linear landscape features, such as fences, trails, and ditches, compared to their resource selection patterns. It appeared that pigs used fence lines, trails and ditches as corridors to transit between habitats, leading to encounters. In addition to transit corridors, some large ditches in the FL site may contain water after major rainfall events, thus providing attractive water resources for wild pigs. Several previous studies have suggested preferences for resources closer to linear features by pigs, such as power lines [11] or agricultural edges [36], indicating use of these areas may be resource driven. Similar findings in other systems indicate early-seral vegetation on linear features like seismic lines, pipelines and industrial access roads provide forage for herbivore ungulates, resulting in increased interspecific contact and competition between these species [21].

Differences between contact- and individual-RSF were also found in relation to strongly preferred landscape features including wetlands, water, and food resources (concordant with previous work [30, 38]). Contacts, however, occurred at wetland habitats and water less than expected by chance. In the study systems, animals displayed a tendency to segregate their use of preferred resources, suggesting a potential dominance or monopolization of these resources. This observation implies a resource despotic pattern rather than a free distribution (Harper 1982).

Supplemental feeding can provide wildlife with an abundant and predictable food source on the landscape, leading to changes in their foraging behaviors and population aggregation at the feeding sites [4]. Thus, supplemental food can often facilitate direct contacts among individuals with implications for disease transmission and other ecological interactions. In the TX sites, bait piles significantly attracted wild pigs [17]. In contrast to cattle supplements in FL, contact locations were also biased towards pig bait in TX indicating that these management techniques could affect contact driven ecological processes, like disease transmission [42]. In this case, baiting was used as an attractant for removal strategies, which may reduce disease concerns. *Potential trade-offs of our contact-RSF model and limitations in wild pig systems.*

Similar to habitat selection, which often varies across different populations, landscapes, and years because of spatial heterogeneity and inter-annual changes in environmental suitability [5], a particular landscape feature may also affect contact in different ways across different ecosystems. In the FL site, we found selection was strong for wetlands, but contact was depressed relative to use. However, in the TX site, female wild pigs tended to avoid wetlands, and wetlands were neither selected for nor avoided by males. Also, wetlands did not have a significant impact on female-male and male-male contacts. This is likely due to the spatial representation of wetlands in this ecosystem of limited small, seasonal, or ephemeral wetlands [2]. Similar differences in findings of population, landscape, and seasonal-specific resource selection have been suggested in other species [3, 25]. Thus, scaling up local contact-RSFs to other landscapes or larger populations requires careful consideration of landscape-specific factors that may affect both resource selection and contact patterns.

Challenges to modeling habitat selection with use-available frameworks are well known, particularly around the definition of availability [14, 16]. Such issues also impact interpretation of outputs from our application of RSFs to contacts. Here, availability was defined as the areas where theoretical contacts between two individuals could occur (i.e., the home range overlap during the overlapped tracking period), which is different from the availability sample used in the individual-RSFs (i.e., the home range during the overlapped tracking period). There are alternative ways to define the availability in the contact-RSF, such as defining it as the union of home ranges. This would enable a scale-wise comparison between contact-RSF and individual-RSF, but logistically, contact could only occur in the home range overlap areas. Investigating how different used-available designs impact contact-RSF interpretation will require further research.

In empirical systems, one limitation is that some between-group contact events might have resulted from mating behaviors rather than resource acquisition. The primary mating season in the FL site seemed to be in August, which is not covered in our study period [7]. In TX, some female wild pigs appeared to be farrowing during the period of this study (N. P. Snow, Personal observation), and subsequently may have isolated themselves from conspecifics during that time. Given that there is a lack of information on timing of reproduction, it was not feasible for us to filter out interactions driven by mating behaviors in the case studies.

## Conclusions

Accurate prediction of the spatial distribution of contacts is valuable for understanding various ecological processes, including disease systems and predator–prey encounters. Our contact-RSF model employs a used-available framework, using contact locations as "used" points and resource select locations as "available" points. This approach enables us to compare the landscape characteristics at the site of contact with those more generally used. Furthermore, it allows us to assess the utility of predicting contact from habitat selection. Our method, therefore, can identify multiple aspects of the spatial ecology of contact-related processes and disentangle their environmental drivers, which opens a door to mechanistic understanding of contact ecology. Application of this approach highlighted that overlaying RSFs of pairs of individuals was not effective at predicting contact locations in two different feral pig systems.

### Supplementary Information

**Additional file 1:** Supplementary Materials for Individual-level patterns of resource selection do not predict hotspots of contact.

## Data Availability

Data will be available upon request.

## Abbreviations

RSF: Resource selection functions
AIC: Akaike information criteria
ABIR: Archbold’s biological station—buck island ranch
NDVI: Normalized difference vegetation index
CTMM: Continuous-time movement model
GPS: Global positioning system
