# Supplementary material for: Individual-level patterns of resource selection do not predict hotspots of contact
Source: Mov Ecol. 2023 Nov 30;11:74. doi: 10.1186/s40462-023-00435-9 (PMC10687890; doi:10.1186/s40462-023-00435-9)
Supplement: Supplementary file 1 — Additional file 1: Supplementary Materials for Individual-level patterns of resource selection do not predict hotspots of contact. [file 40462_2023_435_MOESM1_ESM.docx]

Supplementary Materials for

**Individual-level patterns of resource selection do not predict hotspots of contact**


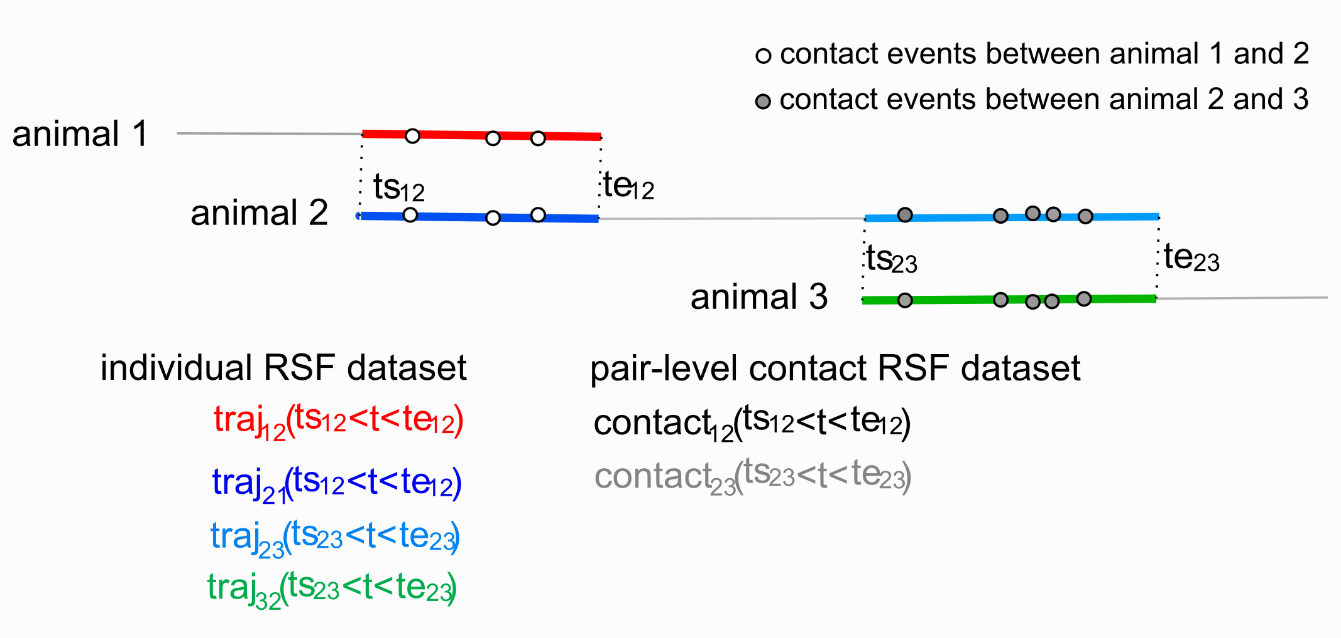


Figure S1. Data reorganization processes for individual- and contact-level RSF models.


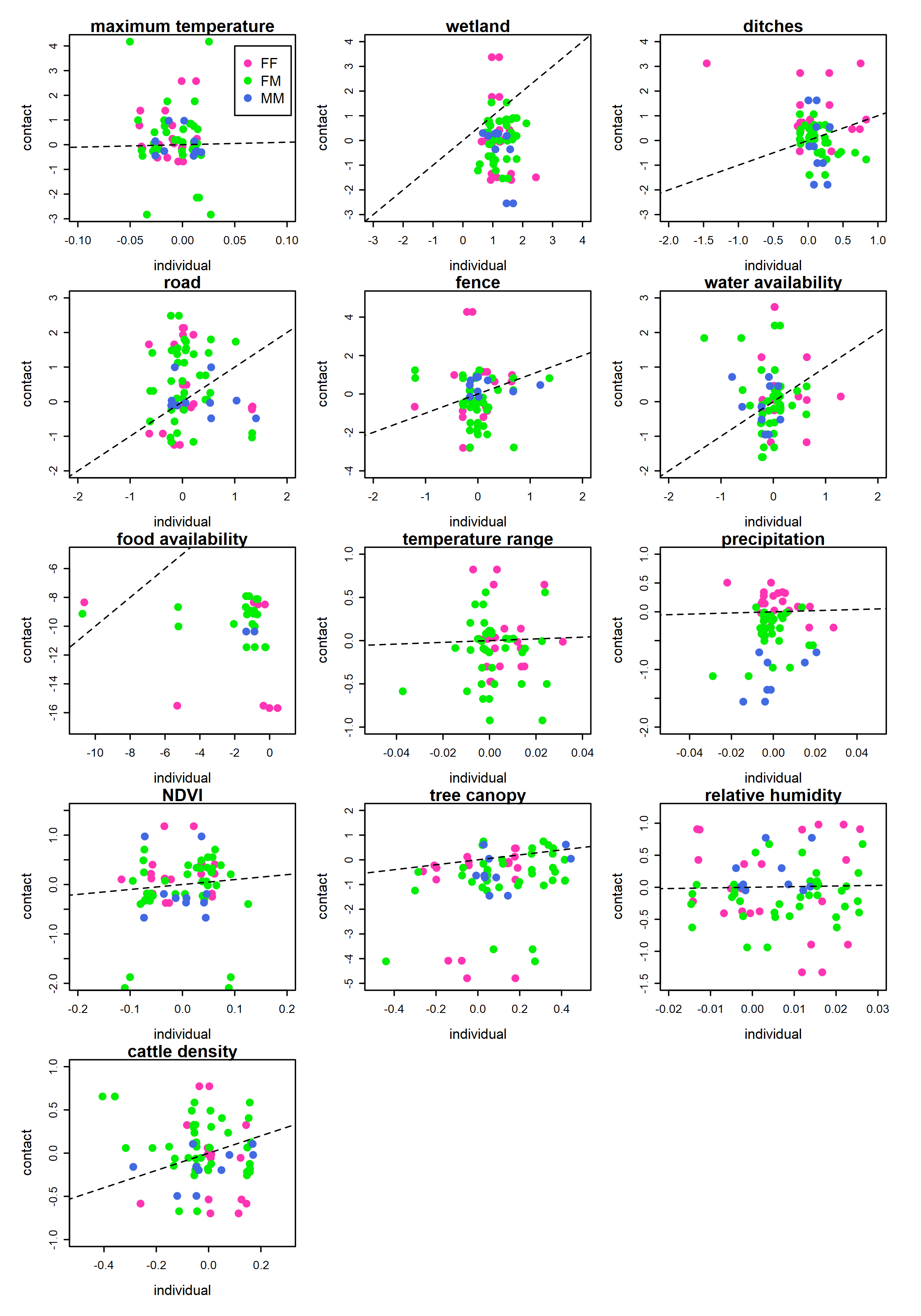


Figure S2. The scatterplot of individual-level and pair-level coefficients for the top selected individual and contact RSF model in FL site. The dashed line is the 1-1 dialog line which indicate the place where the coefficients of individual and contact RSF model are equivalent.


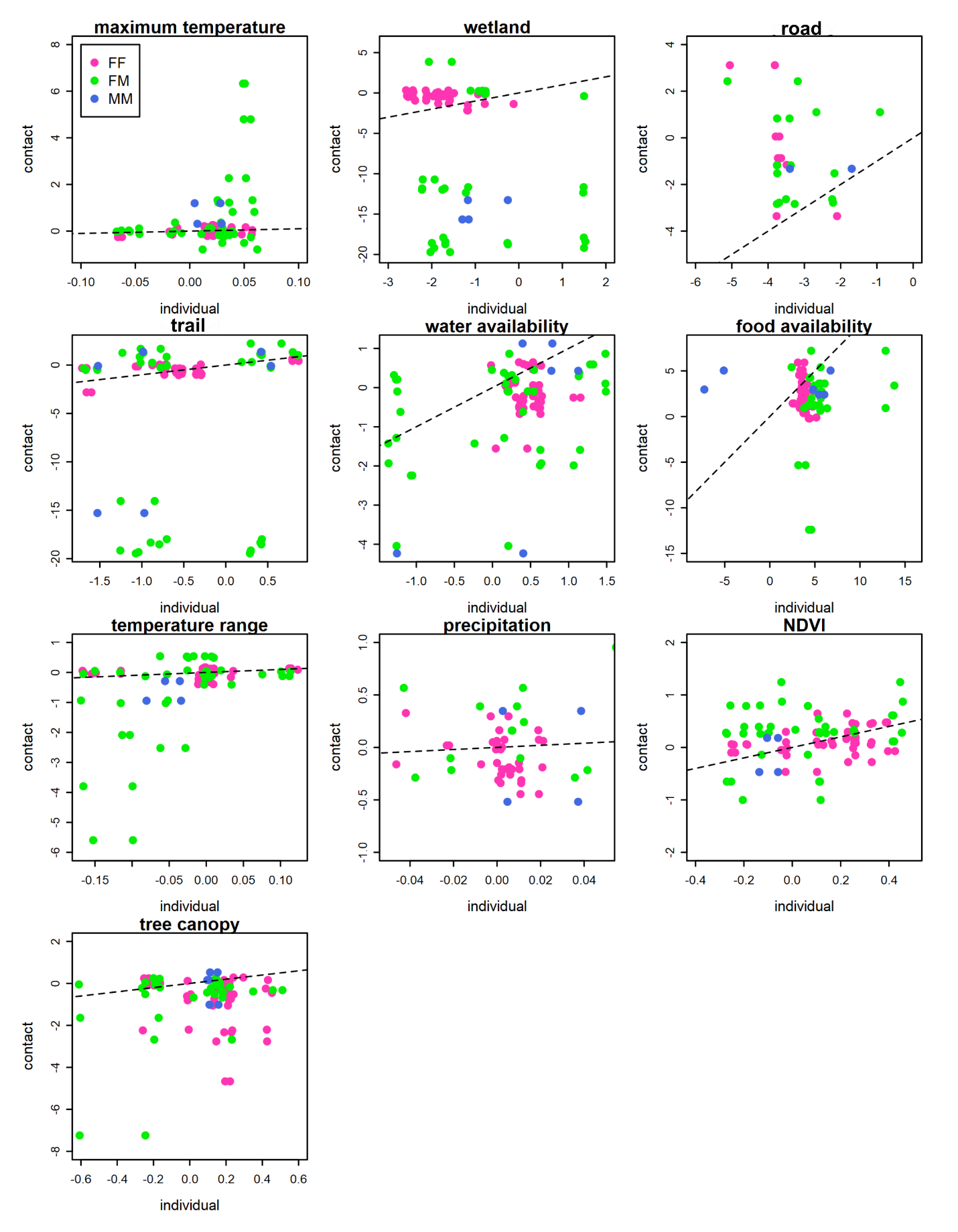


Figure S3. The scatterplot of individual-level and pair-level coefficients for the top selected individual and contact RSF model in Texas site. The dashed line is the 1-1 dialog line which indicate the place where the coefficients of individual and contact RSF model are equivalent.

Table S1. Top five competing models of individual RSF models for F-F, F-M, and M-M pairs at the FL site. The average (and range in parenthesis) of spearman rank correlation coefficients for the top-selected individual- and contact-RSF model were reported.

| No | Model structure | k | ΔAIC | r_s,individual_ | r_s,contact_ |
| --- | --- | --- | --- | --- | --- |
| *F-F candidate models* | | | | | |
| 1.1 | tmax + trange + prcp + ndvi + tree + wetland + rhum + ditch +fence + water + road + food + cattle | 14 | 0 | 0.74 (0.44, 0.94) | 0.67 (0.32, 0.92) |
| 1.2 | tmin + trange + prcp + ndvi + tree + wetland + vp + ditch +fence + water + road + food + cattle | 14 | 6.6 | - | - |
| 1.3 | tmax + trange + ndvi + tree + wetland + rhum + ditch +fence + water + road + food + cattle | 13 | 50 | - | - |
| 1.4 | tmax + trange + prcp + ndvi + tree + wetland + rhum + ditch +fence + water + road + food + cattle | 13 | 223.2 | - | - |
| 1.5 | tmax + trange + prcp + ndvi + tree + wetland + ditch +fence + water + road + food + cattle | 13 | 386.4 | - | - |
| *F-M candidate models* | | | | | |
| 2.1 | tmax + trange + prcp + ndvi + tree + wetland + rhum + ditch +fence + water + road + food + cattle | 14 | 0 | 0.74 (0.34, 0.96) | 0.72 (0.32, 0.93) |
| 2.2 | tmin + trange + prcp + ndvi + tree + wetland + vp + ditch +fence + water + road + food + cattle | 14 | 19.7 | - | - |
| 2.3 | tmax + prcp + ndvi + tree + wetland + rhum + ditch +fence + water + road + food + cattle | 13 | 147 | - | - |
| 2.4 | tmax + trange + ndvi + tree + wetland + rhum + ditch +fence + water + road + food + cattle | 13 | 163.6 | - | - |
| 2.5 | tmax + trange + prcp + ndvi + tree + wetland + ditch +fence + water + road + food + cattle | 13 | 364.3 | - | - |
| *M-M candidate models* | | | | | |
| 3.1 | tmax + prcp + ndvi + tree + wetland + rhum + ditch +fence + water + road + food + cattle | 13 | 0 | 0.78 (0.43, 0.95) | 0.61 (0.31, 0.78) |
| 3.2 | tmax + trange + prcp + ndvi + tree + wetland + rhum + ditch +fence + water + road + food + cattle | 14 | 0.7 | - | - |
| 3.3 | tmax + trange + prcp + ndvi + tree + wetland + vp + ditch +fence + water + road + food + cattle | 14 | 5 | - | - |
| 3.4 | tmax + trange + ndvi + tree + wetland + rhum + ditch +fence + water + road + food + cattle | 13 | 18.7 | - | - |
| 3.5 | tmax + trange + prcp + ndvi + tree + wetland + rhum + ditch +fence + water + road + cattle | 13 | 132.8 | - | - |

Table S2. Top five competing models of individual RSF models for F-F, F-M, and M-M pairs at the TX site. The average (and range in parenthesis) of spearman rank correlation coefficients for the top-selected individual- and contact-RSF model were reported.

| No | Model structure | k | ΔAIC | r_s,individual_ | r_s,contact_ |
| --- | --- | --- | --- | --- | --- |
| *F-F candidate models* | | | | | |
| 1.1 | tmax + tmin + prcp + ndvi + tree + wetland + road + trail + water + food | 10 | 0 | 0.69 (0.32, 0.94) | 0.66 (0.35, 0.91) |
| 1.2 | vp + tmin + ndvi + tree + wetland + road + trail + water + food | 9 | 607.7 | - | - |
| 1.3 | rhum + tmin + ndvi + tree + wetland + road + trail + water + food | 9 | 784.3 | - | - |
| 1.4 | vp + prcp+ ndvi + tree + wetland + road + trail + water + food | 9 | 2143.1 | - | - |
| 1.5 | tmax + tmin + prcp + ndvi + tree + wetland + trail + water + food | 9 | 5217.1 | - | - |
| *F-M candidate models* | | | | | |
| 2.1 | tmax + tmin + prcp + ndvi + tree + wetland + road + trail + water + food | 10 | 0 | 0.66 (0.34, 0.96) | 0.64 (0.41, 0.91) |
| 2.2 | vp + tmin + prcp + ndvi + tree + wetland + road + trail + water + food | 10 | 516.2 | - | - |
| 2.3 | rhum + tmin + prcp + ndvi + tree + wetland + road + trail + water + food | 10 | 733.9 | - | - |
| 2.4 | vp + prcp + ndvi + tree + wetland + road + trail + water + food | 9 | 2026.8 | - | - |
| 2.5 | tmax + tmin + prcp + ndvi + tree + wetland + trail + water + food | 9 | 5853.9 | - | - |
| *M-M candidate models* | | | | | |
| 3.1 | tmax + tmin + prcp + ndvi + tree + wetland + road + trail + water + food | 10 | 0 | 0.68 (0.24, 0.93) | 0.57 (0.3, 0.81) |
| 3.2 | vp + tmin + prcp + ndvi + tree + wetland + road + trail + water + food | 10 | 76.5 | - | - |
| 3.3 | rhum + tmin + prcp + ndvi + tree + wetland + road + trail + water + food | 10 | 92.2 | - | - |
| 3.4 | vp + prcp + ndvi + tree + wetland + road + trail + water + food | 9 | 294.9 | - | - |
| 3.5 | tmax + tmin + prcp + ndvi + tree + wetland + trail + water + food | 9 | 1323.8 | - | - |

Table S2. Coefficients of the top-selected individual and contact RSF models under different sex pairs in FL and TX sites.

| Variables | indFF | contactFF | indFM | contactFM | indMM | contactMM |
| --- | --- | --- | --- | --- | --- | --- |
| FL site | | | | | | |
| tmax | -0.008 [-0.015, -0.002] | 0.242 [-0.255, 0.740] | -0.005 [-0.01, -0.001] | -0.006 [-0.044, 0.032] | -0.001 [-0.014, 0.011] | -0.077 [-0.153, 0] |
| prcp | 0.001 [-0.002, 0.005] | -0.109 [-0.279, 0.062] | 0 [-0.004, 0.004] | -0.031 [-0.049, -0.013] | -0.001 [-0.008, 0.006] | 0.146 [-0.209, -0.083] |
| ndvi | -0.005 [-0.025, 0.014] | 0.072 [-0.062, 0.205] | 0.001 [-0.016, 0.018] | -0.091 [-0.633, 0.451] | -0.001 [-0.034, 0.032] | -2.788 [-4.492, -1.083] |
| tree | 0.040 [-0.043, 0.122] | -0.072 [-0.234, 0.091] | 1.230 [1.131, 1.328] | -0.021 [-0.044, 0.002] | 0.129 [0.011, 0.248] | 0.008 [-0.046, 0.063] |
| wetland | 1.289 [1.135, 1.443] | 0.067 [-0.272, 0.407] | -0.004 [-0.091, 0.083] | 0.269 [-0.04, 0.578] | 1.281 [1.056, 1.506] | 0.287 [-0.278, 0.853] |
| water | 0.132 [0, 0.265] | 0.158 [0, 0.316] | 0.28 [0.138, 0.421] | -0.083 [-0.268, 0.092] | -0.198 [-0.401, 0.006] | -0.379 [1.123, 0.364] |
| road | 0.341[0.114, 0.568] | -0.502 [-1.093, 0.089] | -0.039 [-0.723, -0.063] | 0.448 [0.113, 0.782] | 0.343 [-0.078, 0.764] | 0.292 [-0.455, 1.038] |
| food | -0.022 [-0.272, 0.228] | -8.383 [-9.319, -7.447] | -0.004 [-0.091, 0.083] | -9.103 [-9.962, -8.245] | -0.944 [-1.444, -0.445] | -10.285 [-10.296, -10.274] |
| trange | 0.005 [0.002, 0.009] | -0.001 [-0.094, 0.092] | 0.001 [-0.002, 0.004] | -0.028 [-0.066, 0.011] | - | - |
| rhum | 0.004 [-0.001, 0.009] | -0.267 [-0.546, 0.011] | 0.007 [0.003, 0.011] | -0.028 [-0.048, -0.008] | 0.005 [0, 0.01] | 0.01 [-0.024, 0.044] |
| ditch | 0.157 [0.005, 0.308] | 0.591 [0.227, 0.956] | 0.128 [0.067, 0.189] | 0.155 [-0.018, 0.328] | 0.129 [0.057, 0.202] | -0.218 [-0.963, 0.527] |
| fence | -0.163 [-0.345, 0.018] | 0.591 [0.305, 0.878] | 0.01 [-0.095, 0.115] | 0.157 [-0.164, 0.478] | 0.185 [-0.118, 0.488] | 0.686 [0.246, 1.126] |
| cattle | 0.028 [-0.026, 0.083] | 0.26 [0.075, 0.445] | 0 [-0.044, 0.045] | 0.057 [-0.272, 0.386] | -0.013 [-0.112, 0.085] | -0.19 [-1.085, 0.704] |
| Texas site | | | | | | |
| tmax | 0.013 [0.006, 0.019] | -0.007 [-0.072, 0.059] | 0.013 [0.004, 0.021] | 0.204 [-0.116, 0.524] | 0.021 [0.008, 0.034] | 0.396 [-0.385, 1.177] |
| prcp | 0.003 [-0.006, 0.012] | -0.033 [-0.119, 0.054] | 0.009 [-0.001, 0.019] | -0.088 [-0.274, 0.099] | 0.023 [0.007, 0.039] | -0.326 [-1.420, 0.767] |
| ndvi | 0.004 [-0.04, 0.045] | 0.044 [-0.033, 0.121] | -0.028 [-0.077, 0.022] | 0.263 [0.133, 0.392] | -0.078 [-0.137, -0.019] | 0.059 [-0.714, 0.831] |
| tree | 0.143 [0.116, 0.169] | -0.031 [-0.111, 0.049] | 0.158 [0.123, 0.193] | -0.012 [-0.113, 0.088] | 0.103 [0.035, 0.170] | 0.216 [-0.18, 0.612] |
| wetland | -1.426 [-1.574, -1.279] | -0.373 [-0.600, -0.145] | 0.338 [0.062, 0.614] | 0.114 [-0.077, 0.305] | -0.040 [-0.556, 0.476] | -13.294 [-14.122, -12.466] |
| water | 0.258 [0.153, 0.364] | -0.104 [-0.270, 0.061] | 0.373 [0.205, 0.541] | 0.244 [0.065, 0.423] | 0.896 [0.627, 1.164] | 0.532 [-0.259, 1.323] |
| road | -3.346 [-3.55, -3.14] | 0.206 [-1.544, 1.956] | -2.179 [-2.45, -1.908] | 0.821 [-0.266, 1.909] | -1.793 [-2.103, -1.482] | -1.327 [-1.492, -1.162] |
| food | 4.372 [4.198, 4.546] | 2.247 [1.521, 2.972] | 4.910 [4.739, 5.08] | 2.134 [1.490, 2.779] | 5.219 [4.892, 5.546] | 3.282 [0.581, 5.983] |
| tmin | -0.018 [-0.033, -0.003] | -0.006 [-0.056, 0.045] | -0.029 [-0.047, -0.011] | -0.141 [-0.413, 0.132] | -0.048 [-0.073, -0.023] | -0.370 [-1.026, 0.285] |
| trail | -0.023 [-0.174, 0.128] | -0.190 [-0.408, 0.027] | -0.034 [-0.206, 0.137] | 0.826 [0.500, 1.151] | -0.16 [-0.574, 0.254] | -0.091 [-0.101, -0.081] |
